# MicroRNA Expression in Circulating Leukocytes and Bioinformatic Analysis of Patients With Moyamoya Disease

**DOI:** 10.3389/fgene.2022.816919

**Published:** 2022-05-20

**Authors:** Kaijiang Kang, Yuan Shen, Qian Zhang, Jingjing Lu, Yi Ju, Ruijun Ji, Na Li, Jianwei Wu, Bo Yang, Jinxi Lin, Xianhong Liang, Dong Zhang, Xingquan Zhao

**Affiliations:** ^1^ Department of Neurology, Beijing Tiantan Hospital, Capital Medical University, Beijing, China; ^2^ China National Clinical Research Center for Neurological Diseases, Beijing, China; ^3^ Center of Stroke, Beijing Institute for Brain Disorders, Beijing, China; ^4^ Department of Neurosurgery, Beijing Tiantan Hospital, Capital Medical University, Beijing, China; ^5^ Research Unit of Artificial Intelligence in Cerebrovascular Disease, Chinese Academy of Medical Sciences, Beijing, China

**Keywords:** moyamoya disease, stroke, non-coding RNA, microRNA, wnt signaling pathway

## Abstract

**Objective:** MicroRNAs (miRNAs) in exosomes had been implicated differentially expressed in patient with moyamoya disease (MMD), but the miRNAs expression in circulating leukocytes remains unclear. This study was investigated on the differential expression of miRNAs in peripheral leukocytes between MMD patients and healthy adults, and among patients with subtypes of MMD.

**Materials and methods:** A total of 30 patients with MMD and 10 healthy adults were enrolled in a stroke center from October 2017 to December 2018. The gene microarray was used to detect the differential expression profiles of miRNA in leukocytes between MMD patients and controls, and the differentially expressed miRNAs were verified by the method of real-time PCR. The Gene Ontology (GO) and Kyoto Encyclopedia of Genes and Genomes (KEGG) were used to explore the key signaling pathways and possible pathogenesis of MMD.

**Results:** The microarray results showed 12 differentially expressed miRNAs in leukocytes of MMD patients compared with controls (fold change >2.0, *p* < 0.05 and FDR <0.05), of which 8 miRNAs were upregulated (miRNA-142-5p, miRNA-29b-3p, miRNA-424-5p, MiRNA-582-5p, miRNA-6807-5p, miRNA-142-3p, miRNA-340-5p, miRNA-4270), and 4 miRNAs were downregulated (miRNA-144-3p, miRNA-451a, miRNA-486-5p, miRNA-363-3p). The real-time PCR confirmed seven differentially expressed miRNAs (*p* < 0.05), of which 4 miRNAs (miRNA-29b-3p, miRNA-142-3p, miRNA-340-5p, miRNA-582-5p) were upregulated, and 3 miRNAs (miRNA-363-3p, miRNA-451a and miRNA-486-5p) were downregulated. Both GO and KEGG analysis suggested that the Wnt signaling pathway may be involved in the pathogenesis of MMD. In addition, miRNAs were also differentially expressed among patients with subtypes of MMD.

**Conclusion:** This study indicated that miRNAs are differentially expressed in peripheral leukocytes between MMD patients and healthy adults, and among patients with subtypes of MMD. The Wnt signaling pathway is probably involved in the pathogenesis of MMD.

## Introduction

Moyamoya disease (MMD) is a relatively rare cerebrovascular disease associated with recurrent stroke, characterised by progressive stenosis or occlusion of the circle of Willis with growth of pathological collaterals at the base of the brain (Kuroda and Houkin, 2008; Scott and Smith, 2009). The prevalence of MMD have increased around the world, especially in Japan, Korea, and China (Kuriyama et al., 2008; Duan et al., 2012; Chen et al., 2014; Kim et al., 2015; Kim, 2016; Bao et al., 2019). Currently, the etiology and pathogenesis of MMD are still unclear, so there is a lack of reliable molecular biological markers and effective drugs (Kuroda and Houkin, 2008; Scott and Smith, 2009; Bang et al., 2016). The incidence of MMD has been demonstrated as racial propensity and familial clustering, and some genes have been indicated to be abnormally expressed, which suggests that the MMD may be closely associated with gene expressions (Kuroda and Houkin, 2008; Scott and Smith, 2009; Duan et al., 2018; Wang et al., 2020a; Wang et al., 2021). MMD can be manifested as ischemic, hemorrhagic, or asymptomatic, and hemorrhagic stroke is one of the main factors leading to acute death and severe disability in patients with MMD (Kim et al., 2017; Kang et al., 2019). However, the mechanisms and predictors of hemorrhagic stroke associated with MMD are still limited to the vascular or hemodynamic characteristics, while the underlying pathophysiological mechanisms and biological processes are still unclear (Morioka et al., 2003; Kuroda et al., 2013; Liu et al., 2016; Takahashi et al., 2016).

MicroRNA (miRNA) is a kind of endogenous small RNA with a length of about 18–24 nucleotides, which plays an important regulatory role in the normal metabolism of cells and the development of diseases by inhibiting the translation of messenger RNA (mRNA) into proteins or promoting the degradation of mRNA (Dolz et al., 2017; Mori et al., 2019). Although miRNA has been investigated for several years, its relationship with MMD remains to be elucidated. It has been indicated that miRNAs were abnormally expressed in peripheral blood and cerebrospinal fluid of patients with MMD (Park et al., 2012; Dai et al., 2014; Zhao et al., 2015; Wang et al., 2020b). However, previous studies had mostly focused on the differences of miRNAs in exosomes, which cannot fully reflect the actual expression of intracellular miRNAs. Also, studies have shown that some mononuclear cells in peripheral blood can differentiate into vascular endothelial progenitor cells and vascular smooth muscle progenitor cells, and the pathophysiological process of MMD is often accompanied by inflammatory changes involving a variety of cytokines, suggesting that MMD may be closely associated with peripheral leukocytes (Jung et al., 2008; Kang et al., 2014).

In this study, we investigated the differential expression of miRNA in peripheral leukocytes between MMD patients and healthy adults, and among patients with subtypes (hemorrhagic, ischemic, and asymptomatic) of MMD, to explore the pathogenesis of MMD, and provide the basis for clinical diagnosis, outcome prediction and therapeutic strategy of MMD.

## Materials and Methods

### Study Population

The study was performed according to the guidelines from the Helsinki Declaration, and was approved by the Research Ethics Committee of the hospital. Written informed consent was obtained from all the subjects or their legally authorized representatives. A total of 30 patients, who were diagnosed with MMD in our stroke center from October 2017 to December 2018, were consecutively included in this study. The inclusion criteria included: 1) the age was 18–65 years; 2) diagnosed as MMD according to the 2012 Japanese Moyamoya disease diagnosis and treatment guidelines (Rcotpatosootco, 2012); 3) the patient’s informed consent to the study protocol. The exclusion criteria included: 1) moyamoya syndrome indicated by clinical manifestations, high-resolution MRI, or laboratory examinations; 2) with history of stroke in the past 3 months; 3) patients who had received revascularization surgery; 4) patients with immunological diseases, tumors, or pregnancy. In addition, 10 healthy adults with matching gender and age were included as normal controls.

### Sample Collection and RNA Extraction

Peripheral venous blood samples (about 6 ml) were taken from the antecubital vein into an anticoagulant drying tube under fasting conditions. The whole blood was centrifuged at 2,600 rpm for 10 min at room temperature within an hour. The white blood cells were separated from the middle layer, and the mixed red blood cells were removed with red blood cell lysis buffer. A kind of RNA stabilizer (RNAlater, Thermo Fisher Scientific, Baltics UAB, Vilnius, Lithuania) was added to the cryotube containing white blood cells and was stored at 4°C for 24 h, then stored in −80°C refrigerator for standby. All the samples (from MMD patients and controls) were collected and processed in the same way. Trizol Reagent (Invitrogen, Carlsbad, CA, United States) was used for leukocyte denaturation. The miRNeasy Mini Kit (Qiagen p/n 217004) was used for RNA collection and purification according to the manufacturer’s protocol. The quality evaluation of the total RNA was performed using the Agilent 2100 Bioanalyzer (Agilent, Santa Clara, CA, United States).

### MicroRNA Microarray

The Agilent Human (8*60K) V21.0 miRNA microarrays (Agilent, Santa Clara, CA, United States) were performed using a Gene Expression Hybridization Kit (Agilent’s miRNA Complete Labeling and Hyb Kit-p/n5190-0456) according to the manufacturer’s instructions. Slides were washed in staining dishes with a Gene Expression Wash Buffer Kit (Agilent, Santa Clara, CA, United States) and scanned by an Agilent Microarray Scanner with default settings according to the manufacturer’s instructions. The data was extracted using an Agilent Feature Extraction (AFE) software (version 10.7.1.1), and the raw data were normalized by Quantile algorithm using Gene Spring Software 12.6 (Agilent Technologies). Differentially expressed miRNAs were identified through the filtering of fold-change (>2), *p* value (<0.05) and FDR (<0.05) using R software (version 3.2.3) with the samr package. Also, the differentially expressed miRNAs should not have probes with flag A in at least one group.

### Real-Time Polymerase Chain Reaction

Several differentially expressed miRNAs were quantified by using real-time polymerase chain reaction (RT-PCR) based on the Taqman method. A total of 100 ng of purified RNA was reversely transcribed to cDNA using the Taqman MicroRNA Reverse Transcription Kit (ABI, 4366597) and the stem-loop primers in TaqMan MicroRNA Assays (ABI, United States) according to the manufacturer’s instructions. The RT-PCR was performed using QuantStudio 5 Real-Time PCR System (ABI, United States) and QuantStudio™ Design & Analysis Software with the Taqman Universal PCR Master Mix (ABI, 4440049). The program was as follows: 95°C, 10 min; (95°C, 15 s; 60°C, 1 min) 40 cycles. Expression threshold for each miRNA detector was automatically determined. The homogenous miRNA-16 was used as an internal reference for the analysis. The relative expression level of each miRNA was calculated as fold change adopting the 2−ΔΔCt method. All RT-PCR analyses were performed in triplicate to test the reproducibility.

### Receiver Operating Characteristic Curve Analysis

To assess the potential diagnostic value of identified miRNAs for MMD and subtypes of MMD, receiver operating characteristic (ROC) curve analysis of individual miRNA and combined model of multiple miRNAs was conducted.

### Bioinformatics Analysis

In this study, we used TargetScan (http://www.targetscan.org/, TargetScanHuman 7.2) to identify the targets of differentially expressed miRNAs. We defined the predicted target genes by no less than 10 differentially expressed miRNAs as potential functional targets in MMD. To determine the biological relationship between the potential miRNA target genes, the Gene Ontology (GO) and Kyoto Encyclopedia of Genes and Genomes (KEGG) were performed using DAVID Bioinformatics Resources (http://david.abcc.ncifcrf.gov/, DAVID Bioinformatics Resources 6.8). The significance threshold was set to 0.05 in our enrichment analysis.

### Statistical Analysis

The statistical analysis was performed using SPSS (version 22.0, IBM-SPSS, Chicago, IL, United States) and R software (version 3.2.3). Genes with a two-sided *p* value of <0.05 and fold change >2.0 were regarded as statistically significant genes. ROC curve analysis of individual miRNA and combined model of multiple miRNAs was conducted to assess the potential diagnostic value of identified miRNAs for MMD.

## Results

### Demographic Information and Clinical Characteristics

The study recruited 30 patients with MMD (including 10 hemorrhagic MMD patients, 12 ischemic MMD patients, and 8 asymptomatic MMD patients), and 10 healthy adults with matched demographic characteristics. The detailed characteristics of the included subjects were shown in Table 1.

**TABLE 1:** Demographic and clinical characteristics of the subjects.

ID	Subtypes	Gender	Age (years)	Suzuki stage	
				Left	Right
HM_01	Hemorrhagic MMD	Female	32	4	4
HM_02	Hemorrhagic MMD	Female	43	2	3
HM_03	Hemorrhagic MMD	Female	24	3	3
HM_04	Hemorrhagic MMD	Female	49	6	6
HM_05	Hemorrhagic MMD	Male	49	6	3
HM_06	Hemorrhagic MMD	Male	42	5	5
HM_07	Hemorrhagic MMD	Female	50	3	3
HM_08	Hemorrhagic MMD	Female	34	3	3
HM_09	Hemorrhagic MMD	Male	50	0	3
HM_10	Hemorrhagic MMD	Female	42	4	4
IM_01	Ischemic MMD	Male	34	3	3
IM_02	Ischemic MMD	Female	36	3	1
IM_03	Ischemic MMD	Female	46	3	3
IM_04	Ischemic MMD	Male	35	3	3
IM_05	Ischemic MMD	Female	30	4	3
IM_06	Ischemic MMD	female	46	3	3
IM_07	Ischemic MMD	Male	47	4	3
IM_08	Ischemic MMD	Female	21	2	3
IM_09	Ischemic MMD	Female	29	2	2
IM_10	Ischemic MMD	Male	39	5	4
IM_11	Ischemic MMD	Male	37	2	2
IM_12	Ischemic MMD	Male	39	2	2
AM_01	Asymptomatic MMD	Male	42	2	2
AM_02	Asymptomatic MMD	Male	34	5	3
AM_03	Asymptomatic MMD	Female	29	3	3
AM_04	Asymptomatic MMD	Female	38	3	3
AM_05	Asymptomatic MMD	Female	34	4	4
AM_06	Asymptomatic MMD	Female	48	2	2
AM_07	Asymptomatic MMD	Male	50	3	3
AM_08	Asymptomatic MMD	Female	33	6	5
C_01	Control	Male	42	-	-
C_02	Control	Male	35	-	-
C_03	Control	Male	35	-	-
C_04	Control	Male	34	-	-
C_05	Control	Female	34	-	-
C_06	Control	Female	32	-	-
C_07	Control	Female	33	-	-
C_08	Control	Female	35	-	-
C_09	Control	Female	31	-	-
C_10	Control	Female	33	-	-

HM, hemorrhagic moyamoya disease; IM, ischemic moyamoya disease; AM, asymptomatic moyamoya disease; C, controls.

### MicroRNA Microarray

**FIGURE 1:**
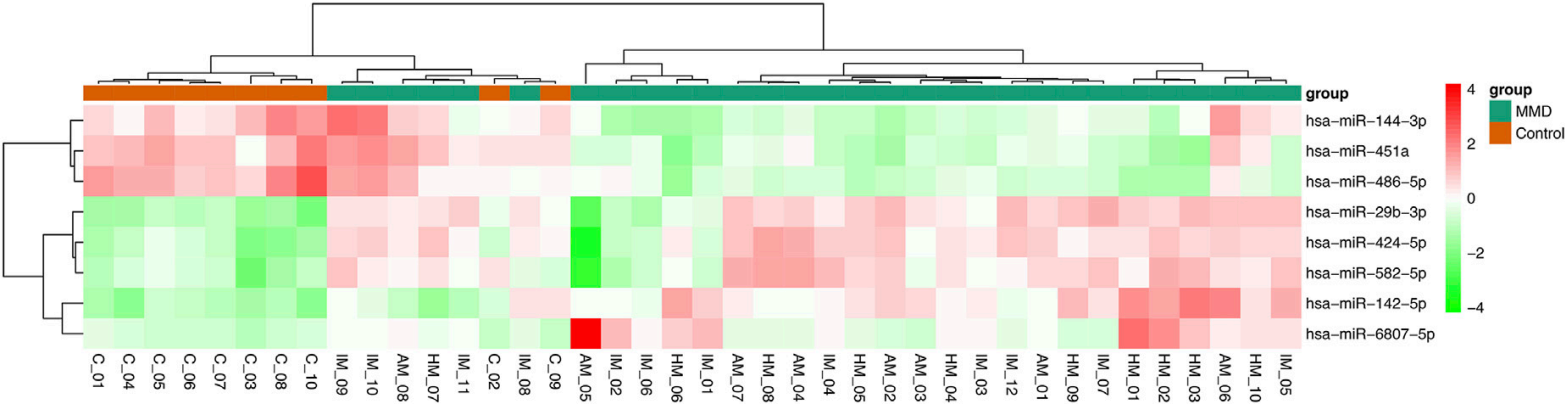
Heat map of differentially expressed miRNAs between MMD patients and healthy controls. In this analysis, 8 miRNAs were found to be differentially expressed, with 5 upregulated (miRNA-142-5p, miRNA-29b-3p, miRNA-424-5p, miRNA-582-5p, miRNA-6807-5p) and 3 downregulated (miRNA-144-3p, miRNA-451a, miRNA-486-5p).

In analysis of 30 MMD patients compared with 10 controls, 8 miRNAs were found to be differentially expressed, with 5 upregulated (miRNA-142-5p, miRNA-29b-3p, miRNA-424-5p, miRNA-582-5p, miRNA-6807-5p) and 3 downregulated (miRNA-144-3p, miRNA-451a, miRNA-486-5p) (Table 2, Figure 1). In addition, according to the intra-group correlation analysis, there were significant differences in RNA expression in 6 samples (4 in the MMD group and 2 in the control group). After the elimination of these 6 samples, miRNA-142-3p, miRNA-340-5p, miRNA-363-3p, miRNA-4270 were also differentially expressed in MMD patients (miRNA-142-3p, miRNA-340-5p and miRNA-4270 upregulated, while miRNA-363-3p downregulated).

**TABLE 2:** Differentially expressed miRNAs in microarray analysis between MMD patients and controls.

Systematic name	*p* values	FDR	Fold change	Regulation
miRNA-142-5p	0.000020	0.002210	2.020448	Up
miRNA-29b-3p	0.000006	0.001311	2.420699	Up
miRNA-424-5p	0.000043	0.003248	2.309035	Up
miRNA-582-5p	0.001293	0.034648	2.228220	Up
miRNA-6807-5p	0.000010	0.001628	2.227829	Up
miRNA-142-3p^a^	0.000018	0.001291	2.160976	Up
miRNA-340-5p^a^	0.000025	0.001549	2.047370	Up
miRNA-4270^a^	0.000478	0.011271	2.110506	Up
miRNA-363-3p^a^	0.000110	0.004134	0.492900	Down
miRNA-144-3p	0.000958	0.027125	0.489388	Down
miRNA-451a	0.000068	0.004695	0.403396	Down
miRNA-486-5p	0.000165	0.009126	0.181111	Down

a The four miRNAs were found differentially expressed in MMD patients after elimination of the 6 samples, in which miRNA expression was significantly different from others according to the intra-group correlation analysis.

**FIGURE 2:**
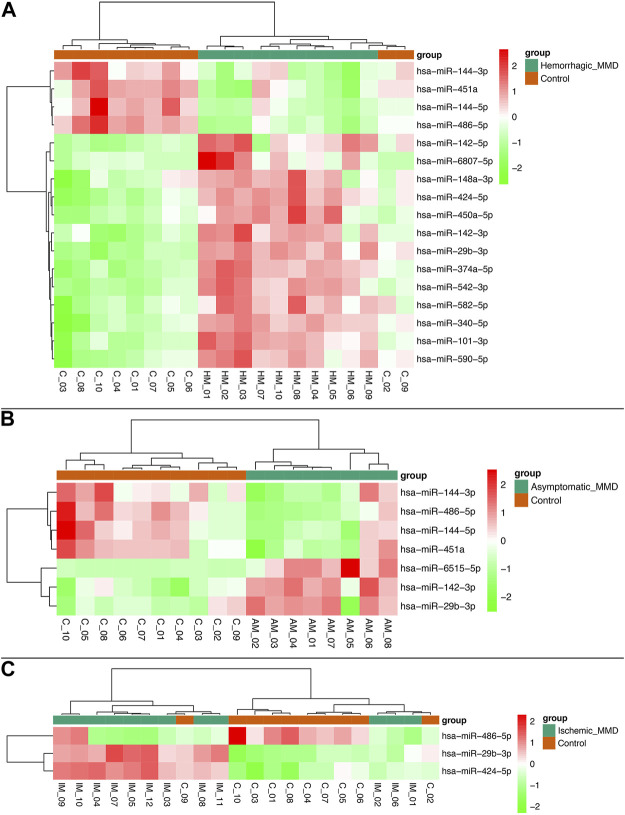
Heat map of differentially expressed miRNAs between subtypes of MMD patients and healthy controls. Seventeen miRNAs were found to be differentially expressed in hemorrhagic MMD patients, with 13 upregulated and 4 downregulated **(A)**. Seven miRNAs were differentially expressed in asymptomatic MMD patients, with 3 upregulated and 4 downregulated **(B)**. Three miRNAs were differentially expressed in ischemic MMD patients, with 2 upregulated and 1 downregulated **(C)**.

In analysis of 10 hemorrhagic MMD patients compared with 10 controls, 17 miRNAs were found to be differentially expressed, with 13 upregulated (miRNA-101-3p, miRNA-142-3p, miRNA-142-5p, miRNA-148a-3p, miRNA-29b-3p, miRNA-340-5p, miRNA-374a-5p, miRNA-424-5p, miRNA-450a-5p, miRNA-542-3p, miRNA-582-5p, miRNA-590-5p, miRNA-6807-5p) and 4 downregulated (miRNA-144-3p, miRNA-144-5p, miRNA-451a, miRNA-486-5p). In analysis of 8 asymptomatic MMD patients compared with 10 controls, 7 miRNAs were found to be differentially expressed, with 3 upregulated (miRNA-142-3p, miRNA-29b-3p, miRNA-6515-5p) and 4 downregulated (miRNA-144-3p, miRNA-144-5p, miRNA-451a, miRNA-486-5p). In analysis of 12 ischemic MMD patients compared with 10 controls, 3 miRNAs were found to be differentially expressed (miRNA-29b-3p and miRNA-424-5p upregulated, while miRNA-486-5p downregulated) (Figure 2).

In addition, miRNA-486-5p was found to be differentially downregulated in hemorrhagic MMD patients, compared with ischemic MMD patients. Also, miRNA-6515-5p was found to be differentially upregulated in asymptomatic MMD patients, compared with symptomatic (hemorrhagic and ischemic) MMD patients.

### Real-Time Polymerase Chain Reaction

**FIGURE 3:**
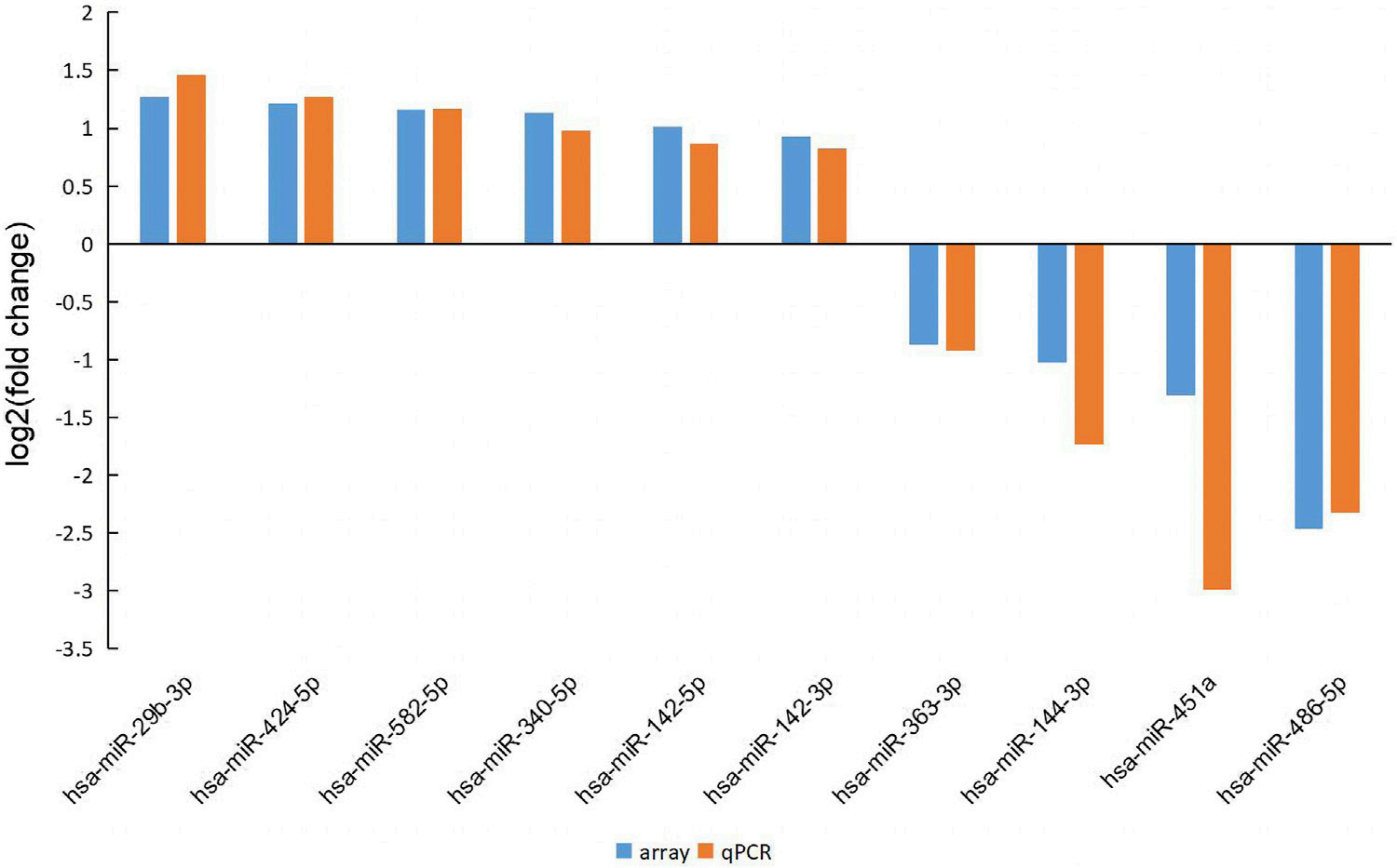
The fold change in the microarray and RT-PCR for all the validated miRNAs. The results demonstrated good consistency between the microarray and RT-PCR.

The RT-PCR verification of 10 differentially expressed miRNAs was performed between 30 MMD patients and 10 controls, including miRNA-142-5p, miRNA-29b-3p, miRNA-424-5p, miRNA-582-5p, miRNA-144-3p, miRNA-451a, miRNA-486-5p, miRNA-142-3p, miRNA-340-5p, miRNA-363-3p. The other two miRNAs (miRNA-4270 and miRNA-6807-5p) were not validated because the potential functional targets had not been well established. The probe was synthesized by Thermo Fisher Scientific, and the sequences of the miRNAs in RT-PCR is shown in Table 3. All RT-PCR analyses were performed in triplicate, which demonstrated strong reproducibility, and all of the 10 genes were identified successfully. However, two normal control samples had higher overall CT values than other samples, but miRNA-16 (internal reference) was relatively lower, which led to higher overall expression and affected the overall analysis results, so the two samples were removed during the final analysis. The fold change in the microarray and RT-PCR for all the validated miRNAs was shown in Figure 3.

**TABLE 3:** The sequences of the validated miRNAs in real-time PCR.

miRNA	Target sequence of the probe
miRNA-16	UAG​CAG​CAC​GUA​AAU​AUU​GGC​G
miRNA-29b-3p	UAG​CAC​CAU​UUG​AAA​UCA​GUG​UU
miRNA-142-3p	UGU​AGU​GUU​UCC​UAC​UUU​AUG​GA
miRNA-142-5p	CAU​AAA​GUA​GAA​AGC​ACU​ACU
miRNA-144-3p	UAC​AGU​AUA​GAU​GAU​GUA​CU
miRNA-340-5p	UUA​UAA​AGC​AAU​GAG​ACU​GAU​U
miRNA-363-3p	AAU​UGC​ACG​GUA​UCC​AUC​UGU​A
miRNA-424-5p	CAG​CAG​CAA​UUC​AUG​UUU​UGA​A
miRNA-451a	AAA​CCG​UUA​CCA​UUA​CUG​AGU​U
miRNA-486-5p	UCC​UGU​ACU​GAG​CUG​CCC​CGA​G
miRNA-582-5p	UUA​CAG​UUG​UUC​AAC​CAG​UUA​CU

**FIGURE 4:**
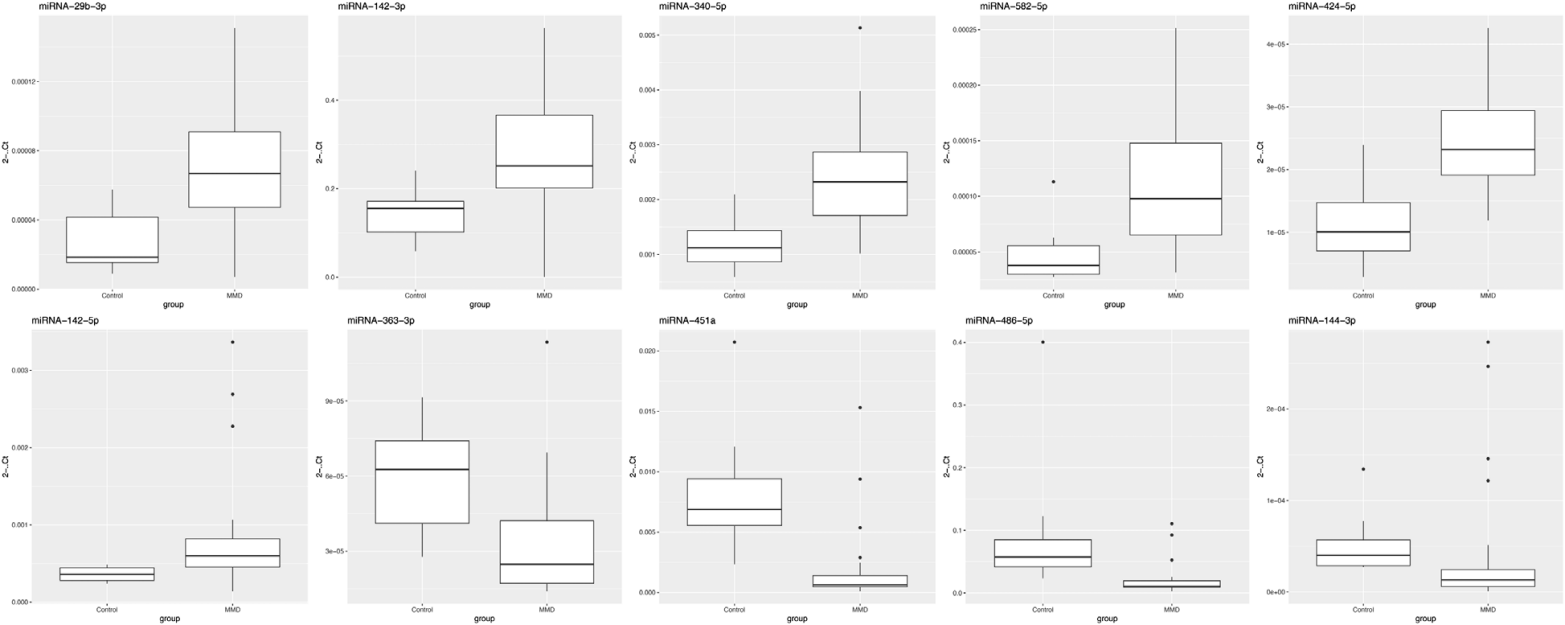
Validation of differentially expressed miRNAs between MMD patients and healthy controls using quantitative real-time PCR. In this analysis, 7 miRNAs had differential expression in MMD patients, of which miRNA-29b-3p, miRNA-142-3p, miRNA-340-5p, miRNA-582-5p were upregulated, while miRNA-363-3p, miRNA-451a, miRNA-486-5p were downregulated.

Compared with 10 healthy adults, 7 miRNAs had differential expression in 30 MMD patients (*p* < 0.05), of which miRNA-29b-3p, miRNA-142-3p, miRNA-340-5p, miRNA-582-5p were upregulated, while miRNA-363-3p, miRNA-451a, miRNA-486-5p were downregulated (Figure 4).

Compared with 10 healthy adults, 9 miRNAs had differential expression in 10 hemorrhagic MMD patients (*p* < 0.05), of which miRNA-29b-3p, miRNA-142-3p, miRNA-142-5p, miRNA-340-5p, miRNA-582-5p were upregulated, while miRNA-451a, miRNA-486-5p, miRNA-144-3p, miRNA-363-3p were downregulated. Compared with 10 healthy adults, 6 miRNAs had differential expression in 8 asymptomatic MMD patients (*p* < 0.05), of which miRNA-29b-3p, miRNA-142-3p, miRNA-340-5p, miRNA-582-5p were upregulated, while miRNA-363-3p, miRNA-451a were downregulated. Compared with 10 healthy adults, 4 miRNAs had differential expression in 12 ischemic MMD patients (*p* < 0.05), of which miRNA-29b-3p, miRNA-142-5p, miRNA-340-5p were upregulated, while miRNA-451a was downregulated.

In addition, the miRNA-142-3p was upregulated in hemorrhagic MMD patients and miRNA-340-5p was upregulated in hemorrhagic and the asymptomatic MMD patients compared with ischemic MMD patients (*p* < 0.05). The miRNA-486-5p was downregulated in hemorrhagic and asymptomatic MMD patients, but there was no statistically significant difference (*p* > 0.05). There was no significant differential expression between the hemorrhagic and the asymptomatic MMD patients.

### Diagnostic Value of Identified MicroRNAs

**FIGURE 5:**
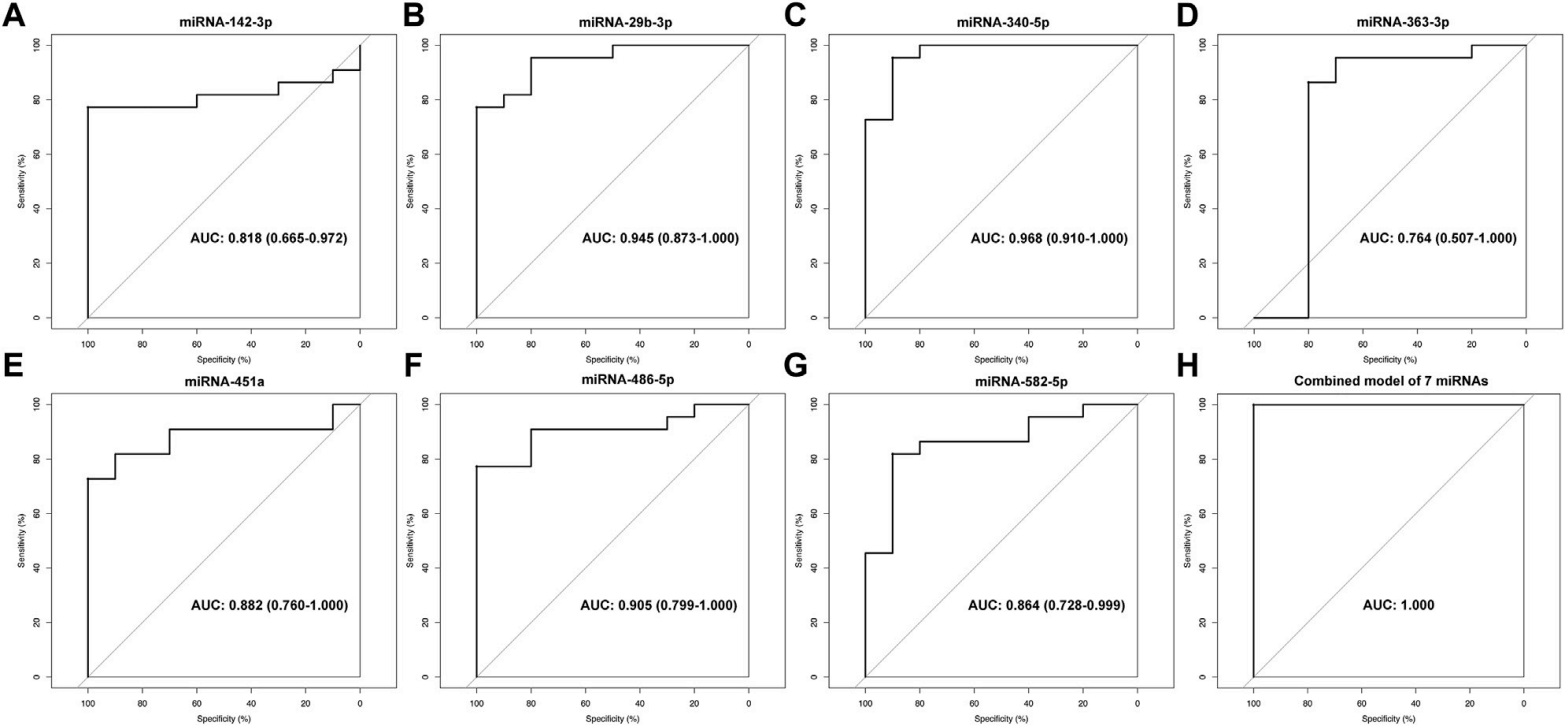
ROC analysis of miRNA-142-3p **(A)**, miRNA-29b-3p **(B)**, miRNA-340-5p **(C)**, miRNA-363-3p **(D)**, miRNA-451a **(E)**, miRNA-486-5p **(F)**, miRNA-582-5p **(G)** and combined model of 7 miRNAs **(H)** for diagnosis of MMD.

To determine the diagnostic values of the significantly differentially expressed miRNAs (miRNA-29b-3p, miRNA-142-3p, miRNA-340-5p, miRNA-582-5p, miRNA-363-3p, miRNA-451a and miRNA-486-5p), ROC curve analysis was performed. The details of area under the curve (AUC) and 95% confidence intervals (95% CI), sensitivity, and specificity of individual miRNA and combined model for diagnosis of MMD can be found in Table 4 and Figure 5.

**TABLE 4:** The details of the AUC, sensitivity, and specificity of each identified miRNA and combined model for diagnosis of MMD.

miRNAs	Disease entity	AUC (95% CI)	Sensitivity (%)	Specificity (%)
miRNA-142-3p	MMD	0.818 (0.665–0.972)	77.3	100.0
miRNA-29b-3p	MMD	0.945 (0.873–1.000)	77.3	100.0
miRNA-340-5p	MMD	0.968 (0.910–1.000)	95.5	90.0
miRNA-363-3p	MMD	0.764 (0.507–1.000)	86.4	80.0
miRNA-451a	MMD	0.882 (0.760–1.000)	72.7	100.0
miRNA-486-5p	MMD	0.905 (0.799–1.000)	77.3	100.0
miRNA-582-5p	MMD	0.864 (0.728–0.999)	81.8	90.0
Combined model of 7 miRNAs	MMD	1	100	100
Combined model of 7 miRNAs	Hemorrhagic MMD	1	100	100
Combined model of 7 miRNAs	Asymptomatic MMD	1	100	100
Combined model of 7 miRNAs	Ischemic MMD	1	100	100

AUC(95%CI), area under the curve (95% confidence interval).

### Target Gene Prediction and Bioinformatics Analysis

**FIGURE 6:**
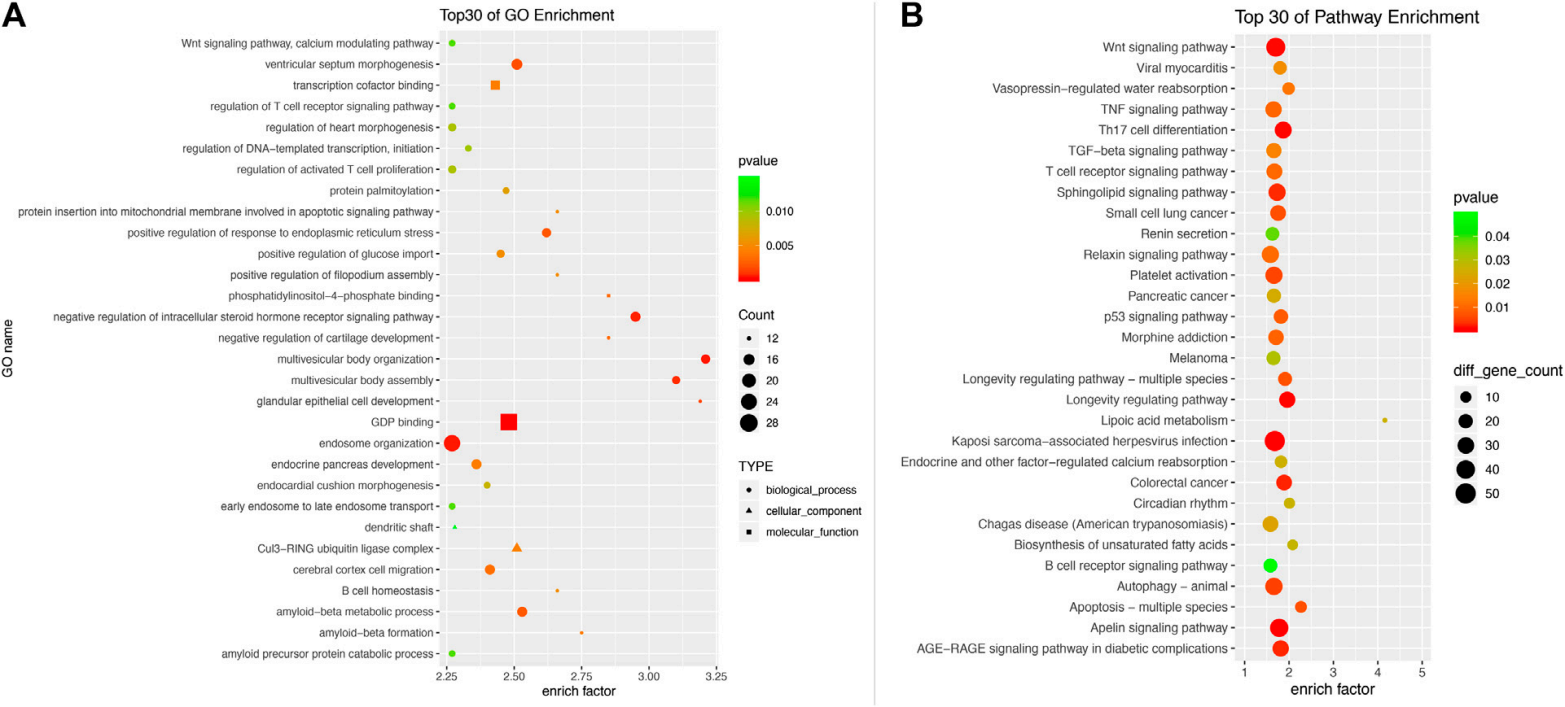
Gene ontology analysis **(A)** and KEGG Pathway analysis **(B)** of 3,728 differentially expressed mRNAs. The associated biological processes and signal pathways were screened according to the criteria (*p* value ≤0.05, number of target genes ≥10) and sequenced according to the enrichment degree. The top 30 biological processes and signal pathways are demonstrated. Both the GO and KEGG analysis suggested that the Wnt signaling pathway may be involved in the pathogenesis of MMD.

**FIGURE 7:**
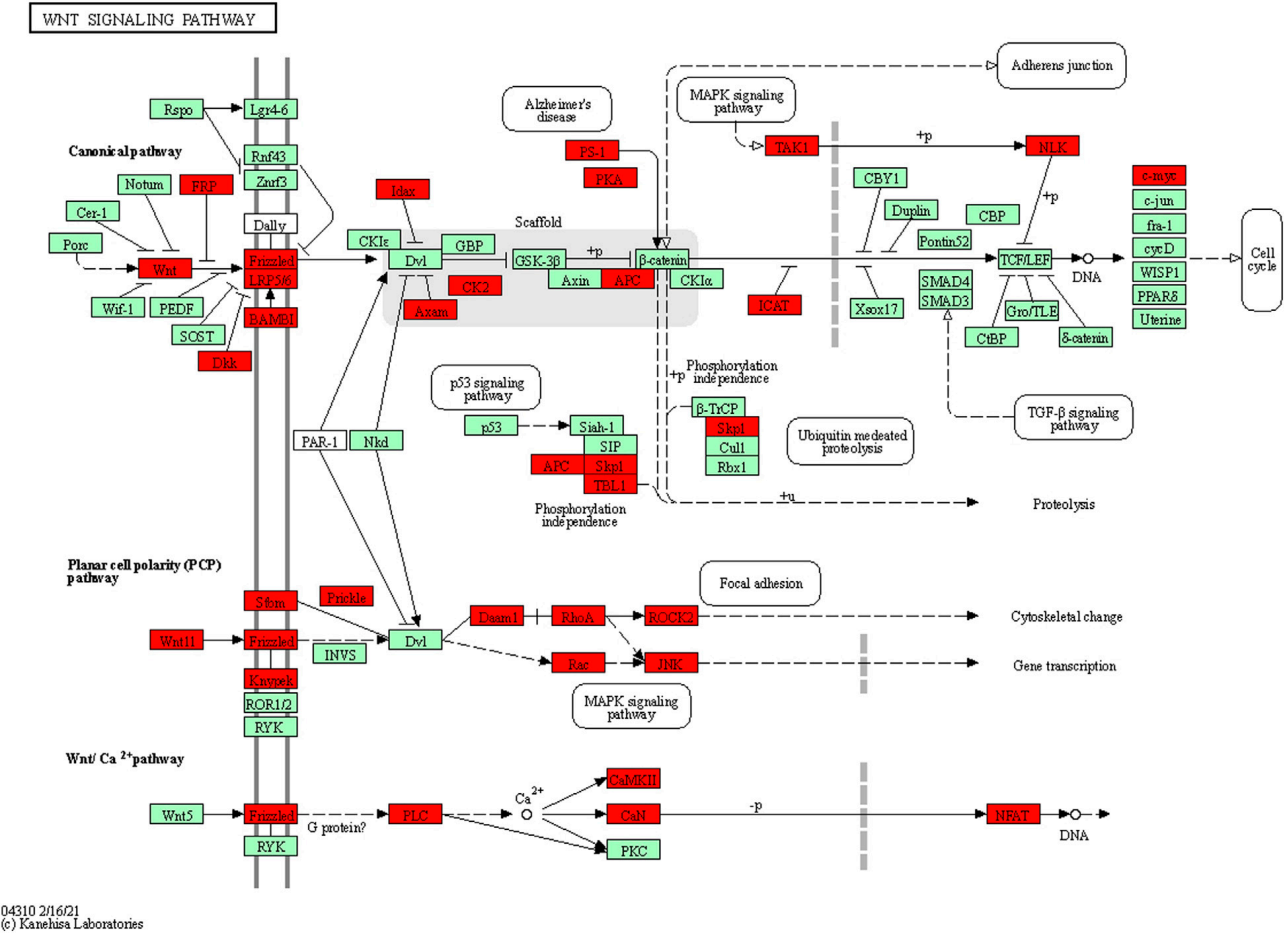
Target genes in the KEGG Wnt pathway map generated using DAVID software. The results indicated that 35 genes (highlighted in red) in the Wnt pathway may be associated with seven differentially expressed miRNAs.

We used the TargetScan target gene prediction program and miRTarBase program to predict the potential target genes of differentially expressed miRNAs by microarray and RT-PCR. According to the seven differentially expressed miRNAs in peripheral leukocytes of MMD patients, a total of 3,728 target genes were predicted based on the TargetScan program, and 63 target genes were predicted based on TargetScan program and miRTarBase program (Table 5). Of the 3,728 target genes, the biological processes and signal transduction pathways that may be involved in the enrichment analysis are carried out through GO and KEGG. The associated biological processes and signal pathways were screened (according to the criteria: *p* value ≤0.05, number of target genes ≥10) and sequenced according to the enrichment degree, and the top 30 biological processes and signal pathways are shown in Figure 6. Both the GO and KEGG analysis suggested that the Wnt signaling pathway may be involved in the pathogenesis of MMD (Figures 6, 7).

**TABLE 5:** Target genes of identified miRNAs based on TargetScan program and miRTarBase program.

miRNAs	Target genes
miRNA-142-3p	BOD1, PROM1, PTPN23, HMGA1, ARNTL, LPP, EGR2, CCNT2, RAC1, ROCK2, TGFBR1, LRRC32, TAB2, HSPA1B, APC, HMGB1
miRNA-29b-3p	PPP1R13B, AQP4, ITGA6, AKT2, LOX, HDAC4, DUSP2, DNMT3B, LRP6, SERPINH1, NKIRAS2, LAMC1, COL3A1, BACE1, COL4A1, ADAM12, PPIC, DNMT3A
miRNA-340-5p	RHOA, IL4, CCNG2
miRNA-363-3p	S1PR1, BCL2L11, FBXW7
miRNA-451a	ADAM10, OXTR, MIF, CPNE3, CAB39, IL6R, MAP3K1, RAB5A, RAB14, CDKN2D, TSC1
miRNA-486-5p	SMAD2, FOXO1, PIK3R1, OLFM4, CDK4, DOCK3, PTEN, ARHGAP5, CIT
miRNA-582-5p	CASP3, CREB1, RAB27A

## Discussions

The results of this study suggested that miRNA-29b-3p, miRNA-142-3p, miRNA-340-5p, and miRNA-582-5p were significantly upregulated, while miRNA-363-3p, miRNA-451a, miRNA-486-5p were significantly downregulated in peripheral leukocytes of patients with MMD compared with healthy controls. In addition, miRNAs were also differentially expressed among patients with subtypes of MMD.

Previous studies had suggested that miRNA-106b, miRNA-130a, miRNA-126, miRNA-125-3p, miRNA-196a, let-7c were abnormally expressed in the peripheral serum of MMD patients (Park et al., 2012; Dai et al., 2014; Zhao et al., 2015). Also, the miRNA-3679-5p, miRNA-6165, miRNA-6760-5p, and miRNA-574-5p had been indicated to be differentially expressed in the cerebrospinal fluid of MMD patients (Wang et al., 2020b). The miRNA-196a was suggested to regulate cell proliferation and apoptosis by regulating the expression of ANXA1 gene in endothelial cells and vascular smooth muscle cells, which may be related to the onset of MMD (Park et al., 2012). It was indicated that the serum let-7c in MMD patients was significantly increased, which can bind to the 3′non-coding region of RNF213 transcribed mRNA, thereby affecting the biological activity of RNF213(21). The RNF213 mutation is currently considered to be a susceptible gene for MMD in Asian populations, which may be involved in the pathogenesis of MMD (Zhang et al., 2016). In the present study, however, these miRNAs did not show abnormal expression in peripheral leukocytes of MMD patients compared with controls, which suggested that there may be significant differences in the expression of miRNA between peripheral leukocytes and serum of patients with MMD. The circulating serum miRNAs reflect only the level of extracellular miRNAs secreted by a variety of cells through exosomes, but cannot fully reflect the true expression of specific intracellular miRNAs, which impeded further investigations on the miRNA-related target genes and cell functions.

The pathological characteristics of MMD are mainly manifested as a gradual degeneration of the smooth muscle in the arterial media, and the abnormal proliferation of the smooth muscle in the arterial intima, leading to progressive stenosis and occlusion of the involved vessels, accompanied by the development of collaterals (Kuroda and Houkin, 2008; Scott and Smith, 2009; Bang et al., 2016). The present study indicated that several biological processes or signal transduction pathways may be involved in the pathophysiological process of MMD, including vascular smooth muscle cell proliferation and metastasis, apoptosis, neovascularization, immune and inflammatory reactions, and thrombosis.

Both the GO analysis and KEGG analysis suggested that the Wnt signaling pathway may be involved in the pathogenesis of MMD (Figures 6, 7). The Wnt signaling pathway is a complex regulatory network consisting of the secreted Wnt protein family, the transmembrane receptor Frizzled family, CK1, Deshevelled, GSK3, APC, Axin, β-catenin, and TCF/Lef family (Mahajan et al., 2012). When the extracellular ligand binds to the cell surface receptor, the intracellular segment of the surface receptor was activated, which transmits extracellular signals into the cells. Previous studies have shown that the Wnt signaling pathway is activated in some cardiovascular and cerebrovascular diseases, and plays an important role in the proliferation, migration, apoptosis, and differentiation of vascular smooth muscle cells, which is one of the mechanisms of MMD (Tsaousi et al., 2011; Foulquier et al., 2018; Shao et al., 2019; Menet et al., 2020). Therefore, the role of the Wnt signaling pathway in MMD is worthy of further investigation.

In addition, miRNAs were also differentially expressed among patients with subtypes of MMD. Patients with MMD need to undergo a chronic conversion of cerebral hemodynamics and feeding arteries, from the original internal carotid artery system to the external carotid artery system or vertebrobasilar artery system. Depending on the degree or weight of vascular occlusion and collateral vasodilation, MMD can be manifested as ischemic, asymptomatic, or hemorrhagic (Kuroda and Houkin, 2008; Scott and Smith, 2009). In this study, the microarray results demonstrated an increasing number of differentially expressed miRNAs from ischemic MMD to asymptomatic MMD to hemorrhagic MMD compared with controls. Also, the real-time PCR results suggested that the miRNA-142-3p was upregulated in hemorrhagic MMD patients, and the miRNA-340-5p was upregulated in hemorrhagic and asymptomatic MMD patients compared with ischemic MMD patients. This indicated that miRNA may be involved in the process of arterial occlusion and collateral development.

In this study, we found that the miRNAs are abnormally expressed in peripheral leukocytes of patients with MMD. A couple of genes had been reported to be mutated in patients with MMD, especially RNF213, which has a high mutation rate in Asian MMD patients (Park et al., 2012; Zhang et al., 2016; Duan et al., 2018; Wang et al., 2020a; Wang et al., 2021). However, the causal relationship and associated mechanism between those mutated genes and MMD is still unclear. In our future studies, we will investigate whether the identified miRNAs are correlated with the mutated genes reported previously. After further verification of real-time PCR with larger sample size, and verification of target genes regulation and cell function regulation, these differentially expressed miRNAs are likely to become new molecular biological markers of MMD and associated stroke, and hold a promise to become new potential targets of therapeutic strategy.

Potential limitations of our studies should be mentioned. First, all of the patients in this study were enrolled from a single center, so potential selection bias may be inevitable. We, however, tried our best to reduce in-house selection bias by collecting patients consecutively. Second, the case-control nature of the study necessitates further prospective cohorts to confirm our conclusions. Third, the sample size of this study was relatively small, and further PCR verification with cross-validation is needed in a large population. Fourth, we used target gene prediction and functional enrichment analysis to indirectly obtain the biological processes that may be involved in the pathogenesis, which lacked verification of miRNA’s regulation of target genes and cell functions, and further vitro cytology experiments are necessitated.

## Conclusion

This study indicated that miRNAs are differentially expressed in peripheral leukocytes between MMD patients and healthy adults, and among patients with subtypes of MMD. The Wnt signaling pathway is probably involved in the pathogenesis of MMD.

## Data Availability

The data presented in the study are deposited in the GEO repository, accession number GSE178501. The datasets can also be found in the article/Supplementary Material.

## References

[B1] Bang O. Y. Fujimura M. Kim S.-K. (2016). The Pathophysiology of Moyamoya Disease: An Update. J. Stroke 18 (1), 12–20. 10.5853/jos.2015.01760 26846756 PMC4747070

[B2] Bao X.-Y. Wang Q.-N. Zhang Y. Zhang Q. Li D.-S. Yang W.-Z. (2019). Epidemiology of Moyamoya Disease in China: Single-Center, Population-Based Study. World Neurosurg. 122, e917–e923. 10.1016/j.wneu.2018.10.175 30404059

[B3] Chen P.-C. Yang S.-H. Chien K.-L. Tsai I.-J. Kuo M.-F. (2014). Epidemiology of Moyamoya Disease in Taiwan. Stroke 45 (5), 1258–1263. 10.1161/strokeaha.113.004160 24676775

[B4] Dai D. Lu Q. Huang Q. Yang P. Hong B. Xu Y. (2014). Serum miRNA Signature in Moyamoya Disease. PloS one 9 (8), e102382. 10.1371/journal.pone.0102382 25093848 PMC4122349

[B5] Dolz S. Górriz D. Tembl J. I. Sánchez D. Fortea G. Parkhutik V. (2017). Circulating MicroRNAs as Novel Biomarkers of Stenosis Progression in Asymptomatic Carotid Stenosis. Stroke 48 (1), 10–16. 10.1161/strokeaha.116.013650 27899750

[B6] Duan L. Bao X.-Y. Yang W.-Z. Shi W.-C. Li D.-S. Zhang Z.-S. (2012). Moyamoya Disease in China. Stroke 43 (1), 56–60. 10.1161/strokeaha.111.621300 22020027

[B7] Duan L. Wei L. Tian Y. Zhang Z. Hu P. Wei Q. (2018). Novel Susceptibility Loci for Moyamoya Disease Revealed by a Genome-wide Association Study. Stroke 49 (1), 11–18. 10.1161/strokeaha.117.017430 29273593

[B8] Foulquier S. Daskalopoulos E. P. Lluri G. Hermans K. C. M. Deb A. Blankesteijn W. M. (2018). WNT Signaling in Cardiac and Vascular Disease. Pharmacol. Rev. 70 (1), 68–141. 10.1124/pr.117.013896 29247129 PMC6040091

[B9] Jung K.-H. Chu K. Lee S.-T. Park H.-K. Kim D.-H. Kim J.-H. (2008). Circulating Endothelial Progenitor Cells as a Pathogenetic Marker of Moyamoya Disease. J. Cereb. Blood Flow Metab. 28 (11), 1795–1803. 10.1038/jcbfm.2008.67 18612318

[B10] Kang H.-S. Moon Y.-J. Kim Y.-Y. Park W.-Y. Park A. K. Wang K.-C. (2014). Smooth-muscle Progenitor Cells Isolated from Patients with Moyamoya Disease: Novel Experimental Cell Model. Jns 120 (2), 415–425. 10.3171/2013.9.jns131000 24160477

[B11] Kang S. Liu X. Zhang D. Wang R. Zhang Y. Zhang Q. (2019). Natural Course of Moyamoya Disease in Patients with Prior Hemorrhagic Stroke. Stroke 50 (5), 1060–1066. 10.1161/strokeaha.118.022771 30909836

[B12] Kim J. S. (2016). Moyamoya Disease: Epidemiology, Clinical Features, and Diagnosis. J. Stroke 18 (1), 2–11. 10.5853/jos.2015.01627 26846755 PMC4747069

[B13] Kim K. M. Kim J. E. Cho W.-S. Kang H.-S. Son Y.-J. Han M. H. (2017). Natural History and Risk Factor of Recurrent Hemorrhage in Hemorrhagic Adult Moyamoya Disease. Neurosurgery 81 (2), 289–296. 10.1093/neuros/nyw179 28402467

[B14] Kim T. Lee H. Bang J. S. Kwon O.-K. Hwang G. Oh C. W. (2015). Epidemiology of Moyamoya Disease in Korea: Based on National Health Insurance Service Data. J. Korean Neurosurg. Soc. 57 (6), 390–395. 10.3340/jkns.2015.57.6.390 26180604 PMC4502233

[B15] Kuriyama S. Kusaka Y. Fujimura M. Wakai K. Tamakoshi A. Hashimoto S. (2008). Prevalence and Clinicoepidemiological Features of Moyamoya Disease in Japan. Stroke 39 (1), 42–47. 10.1161/strokeaha.107.490714 18048855

[B16] Kuroda S. Houkin K. (2008). Moyamoya Disease: Current Concepts and Future Perspectives. Lancet Neurol. 7 (11), 1056–1066. 10.1016/s1474-4422(08)70240-0 18940695

[B17] Kuroda S. Kashiwazaki D. Ishikawa T. Nakayama N. Houkin K. (2013). Incidence, Locations, and Longitudinal Course of Silent Microbleeds in Moyamoya Disease. Stroke 44 (2), 516–518. 10.1161/strokeaha.112.678805 23223508

[B18] Liu P. Han C. Li D.-S. Lv X.-L. Li Y.-X. Duan L. (2016). Hemorrhagic Moyamoya Disease in Children. Stroke 47 (1), 240–243. 10.1161/strokeaha.115.010512 26534975

[B19] Mahajan S. G. Fender A. C. Meyer-Kirchrath J. Kurt M. Barth M. Sagban T. A. (2012). A Novel Function of FoxO Transcription Factors in Thrombin-Stimulated Vascular Smooth Muscle Cell Proliferation. Thromb. Haemost. 108 (1), 148–158. 10.1160/TH11-11-0756 22552808

[B20] Menet R. Lecordier S. ElAli A. (2020). Wnt Pathway: An Emerging Player in Vascular and Traumatic Mediated Brain Injuries. Front. Physiol. 11, 565667. 10.3389/fphys.2020.565667 33071819 PMC7530281

[B21] Mori M. A. Ludwig R. G. Garcia-Martin R. Brandão B. B. Kahn C. R. (2019). Extracellular miRNAs: From Biomarkers to Mediators of Physiology and Disease. Cel Metab. 30 (4), 656–673. 10.1016/j.cmet.2019.07.011 PMC6774861 31447320

[B22] Morioka M. Hamada J.-I. Kawano T. Todaka T. Yano S. Kai Y. (2003). Angiographic Dilatation and branch Extension of the Anterior Choroidal and Posterior Communicating Arteries Are Predictors of Hemorrhage in Adult Moyamoya Patients. Stroke 34 (1), 90–95. 10.1161/01.str.0000047120.67507.0d 12511756

[B23] Park Y. S. Jeon Y. J. Lee B. E. Kim T. G. Choi J.-U. Kim D.-S. (2012). Association of the miR-146aC>G, miR-196a2C>T, and miR-499A>G Polymorphisms with Moyamoya Disease in the Korean Population. Neurosci. Lett. 521 (1), 71–75. 10.1016/j.neulet.2012.05.062 22659075

[B24] Rcotpatosootco Willis. (2012). Guidelines for Diagnosis and Treatment of Moyamoya Disease (Spontaneous Occlusion of the circle of Willis). Neurol. Med. Chir (Tokyo) 52 (5), 245–266. 10.2176/nmc.52.245 22870528

[B25] Scott R. M. Smith E. R. (2009). Moyamoya Disease and Moyamoya Syndrome. N. Engl. J. Med. 360 (12), 1226–1237. 10.1056/nejmra0804622 19297575

[B26] Shao Y. Chen J. Freeman W. Dong L.-J. Zhang Z.-H. Xu M. (2019). Canonical Wnt Signaling Promotes Neovascularization through Determination of Endothelial Progenitor Cell Fate *via* Metabolic Profile Regulation. Stem Cells 37 (10), 1331–1343. 10.1002/stem.3049 31233254 PMC6851557

[B27] Takahashi J. C. Funaki T. Houkin K. Inoue T. Ogasawara K. Nakagawara J. (2016). Significance of the Hemorrhagic Site for Recurrent Bleeding. Stroke 47 (1), 37–43. 10.1161/strokeaha.115.010819 26645256

[B28] Tsaousi A. Williams H. Lyon C. A. Taylor V. Swain A. Johnson J. L. (2011). Wnt4/β-Catenin Signaling Induces VSMC Proliferation and Is Associated with Intimal Thickening. Circ. Res. 108 (4), 427–436. 10.1161/circresaha.110.233999 21193738

[B29] Wang G. Wen Y. Faleti O. D. Zhao Q. Liu J. Zhang G. (2020). A Panel of Exosome-Derived miRNAs of Cerebrospinal Fluid for the Diagnosis of Moyamoya Disease. Front. Neurosci. 14, 548278. 10.3389/fnins.2020.548278 33100957 PMC7546773

[B30] Wang X. Wang Y. Nie F. Li Q. Zhang K. Liu M. (2020). Association of Genetic Variants with Moyamoya Disease in 13 000 Individuals. Stroke 51 (6), 1647–1655. 10.1161/strokeaha.120.029527 32390555

[B31] Wang Y. Yang L. Wang X. Zeng F. Zhang K. Zhang Q. (2021). Meta‐analysis of Genotype and Phenotype Studies to Confirm the Predictive Role of the RNF213 p.R4810K Variant for Moyamoya Disease. Eur. J. Neurol. 28 (3), 823–836. 10.1111/ene.14635 33175469

[B32] Zhang Q. Liu Y. Zhang D. Wang R. Zhang Y. Wang S. (2016). RNF213 as the Major Susceptibility Gene for Chinese Patients with Moyamoya Disease and its Clinical Relevance. J. Neurosurg., 126, 1106–1113. 10.3171/2016.2.JNS152173 27128593

[B33] Zhao S. Gong Z. Zhang J. Xu X. Liu P. Guan W. (2015). Elevated Serum MicroRNA Let-7c in Moyamoya Disease. J. Stroke Cerebrovasc. Dis. 24 (8), 1709–1714. 10.1016/j.jstrokecerebrovasdis.2015.01.041 26070522

